# Muscle activity and hypoalgesia in blood flow restricted versus unrestricted effort‐matched resistance exercise in healthy adults

**DOI:** 10.14814/phy2.16037

**Published:** 2024-07-21

**Authors:** Jinghui Yang, Rory O'Keeffe, Seyed Yahya Shirazi, Sarmad Mehrdad, S. Farokh Atashzar, Smita Rao

**Affiliations:** ^1^ Department of Physical Therapy New York University New York New York USA; ^2^ Department of Electrical and Computer Engineering New York University (NYU) New York New York USA

**Keywords:** blood flow restriction, exercise‐induced hypoalgesia, resistance exercise, sex, surface‐electromyography

## Abstract

This study assessed muscle activity (root mean square, RMS, and median frequency, MDF) to evaluate the acute response to blood flow restriction (BFR) resistance exercise (RE) and conventional moderate intensity (MI) RE. We also performed exploratory analyses of differences based on sex and exercise‐induced hypoalgesia (EIH). Fourteen asymptomatic individuals performed four sets of unilateral leg press with their dominant leg to volitional fatigue under two exercise conditions: BFR RE and MI RE. Dominant side rectus femoris (RF) and vastus lateralis (VL) muscle activity were measured using surface electromyography (sEMG) through exercise. RMS and MDF were calculated and compared between conditions and timepoints using a linear mixed model. Pressure pain thresholds (PPT) were tested before and immediately after exercise and used to quantify EIH. Participants were then divided into EIH responders and nonresponders, and the differences on RMS and MDF were compared between the two groups using Hedges' g. RMS significantly increased over time (RF: *p* = 0.0039; VL: *p* = 0.001) but not between conditions (RF: *p* = 0.4; VL: *p* = 0.67). MDF decreased over time (RF: *p* = 0.042; VL: *p* < 0.001) but not between conditions (RF: *p* = 0.74; VL: *p* = 0.77). Consistently lower muscle activation was found in females compared with males (BRF, RF: *g* = 0.63; VL, *g* = 0.5. MI, RF: *g* = 0.72; VL: *g* = 1.56), with more heterogeneous findings in MDF changes. For BFR, EIH responders showed greater RMS changes (Δ RMS) (RF: *g* = 0.90; VL: *g* = 1.21) but similar MDF changes (Δ MDF) (RF: *g* = 0.45; VL: *g* = 0.28) compared to nonresponders. For MI, EIH responders demonstrated greater increase on Δ RMS (*g* = 0.61) and decrease on Δ MDF (*g* = 0.68) in RF but similar changes in VL (Δ RMS: *g* = 0.40; Δ MDF: *g* = 0.39). These results indicate that when exercising to fatigue, no statistically significant difference was observed between BFR RE and conventional MI RE in Δ RMS and Δ MDF. Lower muscle activity was noticed in females. While exercising to volitional fatigue, muscle activity may contribute to EIH.

## 1 INTRODUCTION

Resistance exercise (RE) confers wide‐ranging health benefits including improved neuromuscular function (Grgic et al., 2019), metabolic function (Pan et al., 2018), and psychological well‐being (Fragala et al., 2019). However, there is significant heterogeneity in the response to RE, and expanded evidence is needed to elucidate mechanisms underlying responses to RE. Recent studies indicate that the extent of muscle activity is a key determinant of RE‐related benefits (Del Vecchio et al., 2019; Siddique et al., 2022). Consequently, RE protocols that enhance muscle activity, either through exercise at high intensity or to task failure, enable substantial gains in muscle size and strength (American College of Sports Medicine, 2009; Morton et al., 2019; Terada et al., 2022). Consistent with this contention, notable gains in neuromuscular function have been reported with high intensity (70%–100% 1 repetition maximum [1RM]) or fatiguing RE protocols (American College of Sports Medicine, 2009; Vieira et al., 2022). However, high intensity RE is also associated with massive mechanical stress on the joints and bones, which limits its applicability in people who are not capable of lifting near‐maximum loads or who are not willing to lift a high load due to the fear of reinjury (kinesophobia) or for whom high loads may be contraindicated, such as in clinical rehabilitation (Flanigan et al., 2013; Loenneke et al., 2012).

Emerging evidence shows that even low intensity RE, which is typically associated with low muscle activation, when combined with blood flow restriction (BFR) can induce comparable or greater muscle size and strength gains compared to moderate‐to‐high intensity conventional RE (Lixandrão et al., 2018). Increased muscle activity (Wernbom et al., 2008; Yasuda et al., 2008) especially the recruitment of high‐threshold motor units during acute exercise (Fatela et al., 2016), and accelerated development of muscle fatigue caused by low intensity BFR are postulated mechanisms underlying the benefits of low intensity RE protocols (Husmann et al., 2018). However, objective evidence comparing muscle activity during low‐to‐moderate intensity RE (15%–50% 1RM) with and without BFR is conflicting (Buckner et al., 2019; Jessee et al., 2018; Kinugasa et al., 2006; Yasuda et al., 2014, 2015). For instance, some studies reported higher muscle activity with conventional RE (15%–30%1RM) compared to intensity matched BFR RE (Buckner et al., 2019; Sousa et al., 2017) while others found contrary evidence showing that BFR‐enhanced muscle activity in low intensity RE (20%–30% 1RM) when compared to conventional RE with matched exercise intensity (Husmann et al., 2018; Yasuda et al., 2013, 2014) or conventional RE with higher exercise intensity (40%–50% 1RM) (Bezerra et al., 2021; Jessee et al., 2018). Furthermore, several studies found no additional effects of BFR on muscle activity in low intensity RE (Freitas, Galletti, et al., 2020; Wilson et al., 2013). These conflicting findings may stem from three key factors: (1) Limited study sample, (2) Inadequate quantification of cuff pressure, and (3) Variability in exercise protocol (e.g., volume‐matched or effort‐matched RE). In terms of study samples, some studies have only included male participants or participants with certain physical activity profiles, and most studies have not assessed habitual exercise in their samples (Bezerra et al., 2021; Buckner et al., 2019; Jessee et al., 2018). Chronic physical activity and habitual exercise can influence muscle fiber type (Wilson et al., 2012), and may contribute to between‐study differences in muscle activity during RE. Second, variability in BFR instrumentation may also contribute to conflicting muscle activity findings. Recent advances in BFR cuff technology have made it possible to accurately quantify cuff pressure and maintain it (“auto regulation”) during the RE protocol (Jacobs et al., 2023). Lastly, some studies have used volume matched RE protocols (e.g., four sets with 30 repetitions in the first set and 15 repetitions in each subsequent set, abbreviated as 30‐15‐15‐15) while others have asked participants to exercise to volitional fatigue (effort‐matched RE protocol). Volume‐matched protocols do not offer personalized training loads and may therefore lead to variable levels of muscle activity during RE protocols. To overcome these limitations, studies with adequate characterization of the sample, personalized cuff pressures in accordance with current BFR application recommendations (Patterson et al., 2019), and personalized training loads are essential.

There is a lack of representation of females in current BFR research (Counts et al., 2018). Specifically, many studies in the similar context were either male subjects only (Bezerra et al., 2021; Cayot et al., 2016; Cerqueira et al., 2021; Cook et al., 2013; Fatela et al., 2018) or with a dominance of male subjects (Jessee et al., 2018; Sousa et al., 2017; Wernbom et al., 2008). A few studies engaged relatively equivalent male and females but not aimed to explore the potential sex differences (Freitas, Galletti, et al., 2020; Gizzi et al., 2021; Jessee et al., 2019). Therefore, the role of sex in BFR‐related muscle activity remains unclear.

In asymptomatic pain‐free individuals, a reduction in pain sensitivity is usually found following exercise (Wewege & Jones, 2020). This phenomenon is termed “exercise‐induced hypoalgesia” (EIH). Similar to muscle size and strength gain, the magnitude of EIH accompanying RE is closely related to exercise intensity and duration, with higher RE intensity and longer exercise duration usually eliciting greater EIH (Hoeger Bement et al., 2008; Koltyn, 2000). Our work (unpublished) and recent evidence shows that, even with very low intensity (30% 1RM), BFR RE can potentially induce considerable hypoalgesia (Hughes & Patterson, 2020; Song et al., 2021). Moreover, though the hypoalgesic effects can be observed in both exercise limb and unexercised limb, the magnitude of EIH is consistently greater in the exercised area compared to non‐exercised area (Hughes & Patterson, 2020; Song et al., 2021), which may indicate the potential impact of local muscular activity in BFR RE‐related pain modulation. To the best of our knowledge, no study has tested the relationship between muscle activity and EIH in BFR RE.

Therefore, the primary objective of this study is to compare the effect of low intensity BFR RE and conventional moderate intensity RE on muscle activity in a single session effort‐matched RE protocol. A secondary goal is to perform an exploratory analysis and examine, first, the effect of sex on muscle activity, and second, the role of muscle activity on exercise‐induced hypoalgesia (EIH). We hypothesize that low intensity BFR RE will induce less changes in muscle activity parameters than moderate intensity RE. We also hypothesized that both sexes would show similar activity and that the extent of muscle activity might impact the magnitude of EIH.

## 2 METHODS

### 2.1 Participants

The study was approved by the New York University Institutional Review Board (IRB‐FY2019‐3039). Participants with mixed training status were recruited using poster flyers. An online survey was used to determine the participants' eligibility. Individuals between 18 and 15 years of age were invited to participate in this study. Participants were excluded if they had major surgery in last 6 months, were diagnosed with cancer in last 6 months, were bedridden for more than 3 days prior to data collection, had peripheral vascular disease or other circulatory issues, or musculoskeletal conditions that may affect RE participation, or ongoing pain conditions or had any conditions that may impact pain modulation. This manuscript focuses on muscle activity measures obtained from 14 asymptomatic individuals (Mean (SD) age = 26 (4) years, Mean (SD) BMI = 22.7 (3.1) kg/m^2^). All participants provided written informed consent prior to participation.

### 2.2 Experimental protocol

The current study employed a cross‐over design. All participants completed two study visits, one for each RE condition (BFR or MI), and the order of the two conditions was counterbalanced, with half of the participants performing BFR RE at their first visit and the other half performing moderate intensity RE at their first visit. The participants refrained from strenuous exercise 48 h prior to their visit. The two visits were scheduled for at least 72 h and no more than 21 days apart. On the first visit, participants' demographic information and current exercise habits were recorded. Leg press 1RM of the dominant leg was assessed using a leg press machine (Inflight Systems, Newport Beach, CA) (Miller et al., 2018) following the guideline proposed by National Strength and Conditioning Association (2021).

### 2.3 RT protocol

The range of motion (ROM) of the knee was set from full extension to 100‐degree flexion (Wernbom et al., 2009). For moderate intensity condition, participants performed the unilateral leg press at 50% 1RM. For BFR RE, participants performed the leg press at 30% 1RM with the cuff inflated to 60% limb occlusion pressure (%LOP, described below). A metronome was used to ensure that participants held the cadence of 1 s concentric contraction and 1 s eccentric contraction. To match the effort in the two experiential conditions, participants performed four sets of resistance exercise in each visit with a protocol of 30‐15‐15 repetitions at the first three sets and exercise to failure at the fourth set. Failure was defined as the inability of completing the exercise in whole range within the 2‐s period. There were 30 s rest between each set. At the end of each set, participants immediately reported their exertion level based on the Borg scale (6–20).

### 2.4 Blood flow restriction

In line with current BFR RE practice (Patterson et al., 2019), LOP was determined in the seated position with participant's dominant leg placed on the leg press (same position as the exercise). A cuff (10 cm width, Smartcuffs, Smart Tools Plus, US) was placed at the most proximal region of the dominant thigh. Utilizing an Ultrasonic Pocket Doppler probe (SD3 Vascular, Edan, China), a distinct and steady pulse was identified at the posterior tibial artery near the lateral malleolus (Laurentino et al., 2020; Masri et al., 2016). The cuff was continuously inflated to the pressure of 50 mmHg, and subsequently increased by 10 mmHg every 10 s until the tibial pulse disappeared, indicating that the total LOP was reached (Vehrs et al., 2023). Following this, the cuff was deflated, and 60% LOP was determined and implemented throughout the BFR RE protocol, including the rest periods between sets.

### 2.5 Outcome measures

#### 2.5.1 Surface electromyography

Muscle activity was collected using wireless bipolar surface electromyography (sEMG). Electrodes were placed on the vastus lateralis and rectus femoris (O'Keeffe, Shirazi, et al., 2023; O'Keeffe, Yang, et al., 2023). Surface electrodes (Trigno, Delsys Inc, USA) were placed on the muscle belly of rectus femoris (50% on the line from the anterior spina iliaca superior to the superior part of the patella) and vastus lateralis (2/3 on the line from the anterior spina iliaca superior to the lateral side of the patella) following *SENIAM* guidelines (Hermens et al., 2000). The skin preparation included shaving, abrasion, and wiping with alcohol wipes. An electrogoniometer (Biometrics Ltd, UK) was positioned at the lateral of the knee to record the knee angles at 296 Hz throughout the exercise protocol (O'Keeffe, Yang, et al., 2023).

#### 2.5.2 Exercise‐induced hypoalgesia (EIH)

Pressure pain threshold (PPT) was used to characterize mechanical pain sensitivity, defined as the minimum pressure a subject recognizes as an experience of pain (Fischer, 1987). PPT was measured before and immediately after exercise in supine. A handheld, digital, pressure algometer with a 1 cm^2^ circular trip (FPIX 100, Wagner Instruments, CT, USA) was used to measure PPT. Evaluating PPT with a handheld algometer has been previously demonstrated to be valid and reliable (Kinser et al., 2009; Mutlu & Ozdincler, 2015). A single tester (JY) applied the algometer at a controlled rate (5 N/s) perpendicular to the skin (Jayaseelan et al., 2020; Naugle et al., 2012; Vaegter et al., 2018). Participants were instructed to indicate when the sensation of pressure became painful. A pilot analysis was performed to evaluate test–retest reliability. In accordance with current evidence (Karasuno et al., 2016; Pearcey et al., 2015), PPT was assessed three times at the midway between the iliac crest and superior border of the patella of the dominant and nondominant limbs, and the mean value was used for analysis. Exercise‐induced hypoalgesia was calculated as the percentage of change of the PPT values pre‐and post‐exercise: EIH = (PPT_post‐_PPT_pre_)/PPT_pre_ (Naugle et al., 2012). Based on the minimal detectable change of PPT reported in a healthy pain free population (4.7 N), participants who showed at least 4.7 N increase in PPT after exercise were identified as EIH responders (Walton et al., 2011). Participants who did not show a 4.7 N increase in PPT or showed a decrease in PPT after exercise (i.e., negative EIH) were classified as nonresponders (Walton et al., 2011).

Exercise volume was calculated as the product of exercise intensity and total repetitions.

RPE was expressed as the average exertion level (6–20) at the end of each set.

Participation in habitual exercise was also assessed using a self‐report questionnaire at the first visit. Participants were classified as regular RE if they participated in strength training at least three times per week in the last 3 months.

### 2.6 Data analysis

The surface electrodes were connected to an amplifier and digitized (Trigno, Delsys Inc, USA). The signal was sampled at a frequency of 1259 Hz and band‐pass filtered at 15–450 Hz. The computer software EMGworks (Delsys Inc, USA) and Microsoft Excel (Microsoft 365, Microsoft Corporation, Redmond, WA, USA) were used to analyze the data. Only the concentric phase contraction data were analyzed. The concentric phase was determined by the electro‐goniometer.

EMG amplitude (Root Mean Square, RMS) was calculated from the average of the first three repetitions of the first set and the last three repetitions of each set. The change in RMS (Δ RMS) due to RE was quantified as the percentage difference from the start of the exercise and the end of the exercise: (RMS_end_ – RMS_start_)/RMS_start_. The first repetition of the first set and the last repetition of the last set were analyzed for median frequency (MDF) changes. The change in MDF (Δ MDF) due to RE was quantified as the percentage difference from the start of the exercise to the end of the exercise: (MDF_start_ – MDF_end_)/MDF_start_.

### 2.7 Statistical analysis

All statistical analysis was conducted with SPSS 22.0 software (SPSS Inc., Chicago, Illinois, US), with variability represented as standard deviation (SD). The normality of data was tested by Shapiro–Wilk test. Paired *t*‐tests or Wilcoxon were used accordingly to compare RPE, repetition, exercise volume, Δ RMS, and Δ MDF in two conditions. A linear mixed model was used to examine the effects of condition (BFR vs. MI) and timepoint (at the start point and the end point of each set) on rectus femoris and vastus lateralis Δ RMS. Bonferroni corrected post hoc *t*‐test were used for multiple pairwise comparisons. A linear mixed model was used to examine the effects of condition (BFR vs. MI) and timepoint (at the start and the end of exercise) on rectus femoris and vastus lateralis Δ MDF. Hedges' g was calculated to quantify the differences in muscle activity between EIH responders and nonresponders (effect size small = 0.15, medium = 0.40, large = 0.75) (Brydges, 2019). Hedges' g was also calculated to quantify the differences in muscle activity between females and males.

## 3 RESULTS

Fourteen asymptomatic participants completed two visits and were included in the analysis (Table 1). There were no statistically significant between‐visit differences observed for 1‐RM (104.6 ± 67.3 and 105.4 ± 66.0 lbs., BFR and MI, respectively, *p* = 0.93), baseline PPT (46.6 ± 19.5 and 45.7 ± 11.9 N, BFR and MI, respectively, *p* = 0.82), and perceived exertion levels across the two RE protocol (17.3 ± 1.4 and 16.7 ± 2.3, BFR and MI, respectively, *p* = 0.45). A significantly smaller number of repetitions and lower total exercise volume were noted with BFR compared to moderate intensity RE (number of repetitions: 50.6 ± 20.1 vs. 63.1 ± 11.5, *p* = 0.01, total exercise volume: 1744 ± 1522 vs. 3199 ± 2233, *p* < 0.001, BFR vs. MI, respectively). The average cuff pressure applied for the BFR condition was 111.9 ± 11.2 mm Hg.

**TABLE 1:** Demographic data for all participants (*M* ± *SD*).

Characteristics	Male	Female
*N*	7	7
Age (years)	23 ± 3	22 ± 9
BMI (kg/m^2^)	20.9 ± 3.5	18.9 ± 7.3
Regular RE (*n*)	1	2

Abbreviation: RE, resistance exercise.

### 3.1 Δ RMS

**FIGURE 1:**
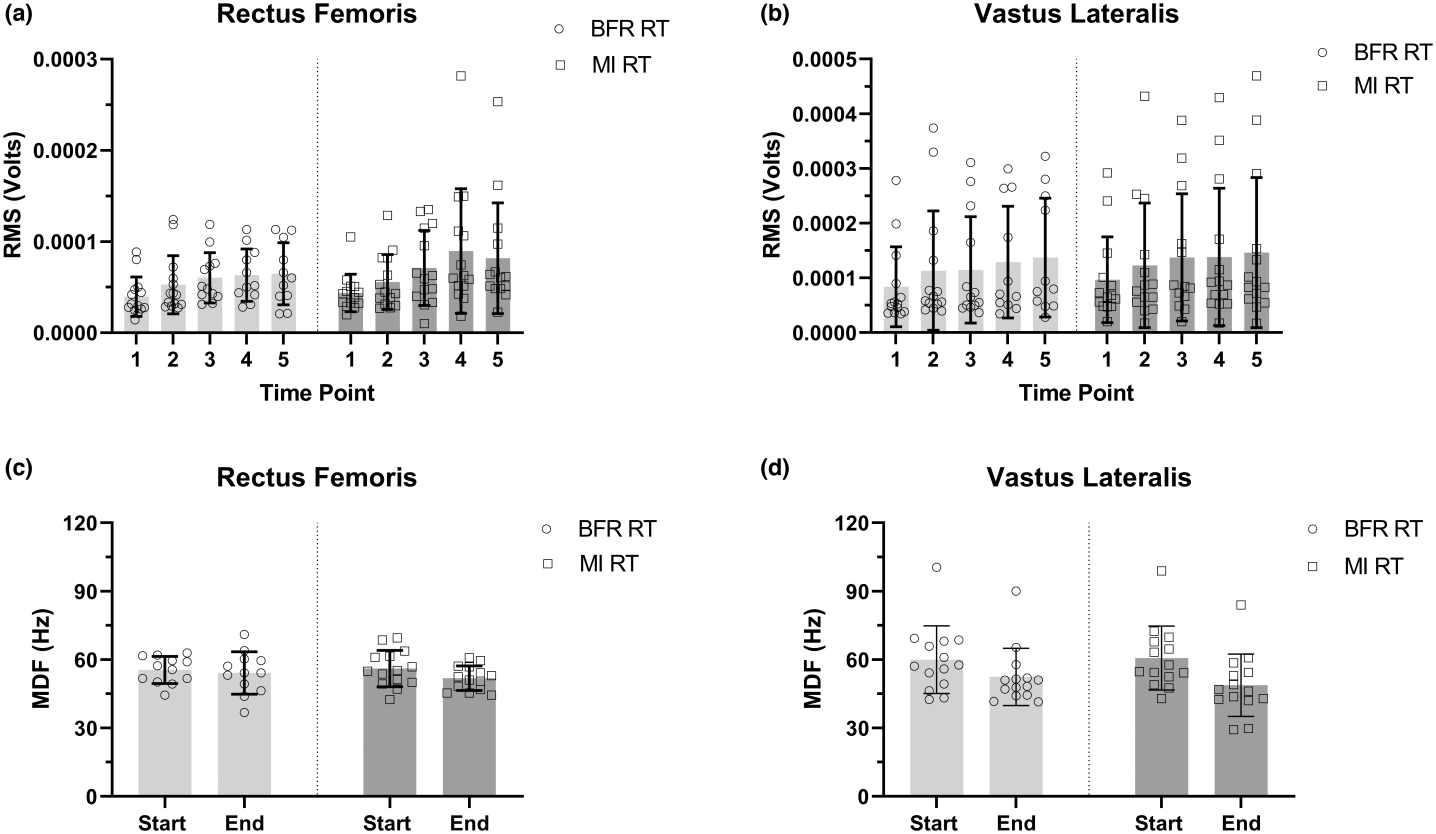
RMS and MDF changes over time and conditions (Mean with SD). Timepoint 1, beginning of the 1st set; 2, end of the 1st set; 3, end of the 2nd set; 4, end of the 3rd set; 5, end of the 4th set. Start, the beginning of the first set; End, the end of the fourth set.(a) RF RMS increased significantly through exercise, with no interaction and main effect of conditions. (b) VL RMS increased significantly through exercise, with no interaction and main effect of conditions. (c) RF MDF decreased significantly through exercise, with no interaction and main effect of conditions. (d) VL MDF decreased significantly through exercise, with no interaction and main effect of conditions.

For rectus femoris, the linear mixed model analysis indicated no significant interaction between time and condition (*F*(4, 91) = 0.72, *p* = 0.58), a significant main effect of time (*F*(1.549, 35.25) = 7.4, *p* = 0.0039) and no significant main effect of condition, (*F*(1, 26) = 0.73, *p* = 0.40) (Figure 1A).

For vastus lateralis, similarly, linear mixed model analysis indicated no significant interaction between time and condition (*F*(4, 91) = 0.23, *p* = 0.92), a significant main effect of time (*F*(1.502, 34.17) = 9.43, *p* = 0.001) and no significant main effect for condition (*F*(1, 26) = 0.19, *p* = 0.67) (Figure 1B). The means and standard deviation for rectus femoris Δ RMS and vastus lateralis Δ RMS are presented in Table 2 below, significant pairwise comparisons are indicated by *.

**TABLE 2:** Rectus femoris and vastus lateralis RMS changes over time—mean, standard deviation, and pairwise comparisons.

Muscle	Condition	Timepoint	*M* (*SD*) (μV)	Percent changes vs. timepoint 1	Pairwise comparison (Main time effect)
RF	BFR RE	1	39.6 (21.6)		1 vs. 2** 1 vs. 3*** 1 vs. 4* 1 vs. 5* 2 vs. 3*
		2	52.6 (31.9)	33.07%	
		3	60.3 (27.6)	52.32%	
		4	62.7 (31.3)	58.47%	
		5	63.4 (37.7)	60.21%	
	MI RE	1	43.5 (20.6)		
		2	55.6 (30.2)	27.70%	
		3	67.6 (40.7)	55.34%	
		4	92.0 (70.5)	111.22%	
		5	81.6 (60.7)	87.48%	
VL	BFR RE	1	83.9 (73.0)		1 vs. 2** 1 vs. 3* 1 vs. 4** 1 vs. 5** 2 vs. 5* 3 vs. 4** 3 vs. 5** 4 vs. 5**
		2	113.4 (108.9)	35.20%	
		3	114.7 (97.2)	36.77%	
		4	122.2 (100.2)	45.69%	
		5	152.3 (115.3)	81.59%	
	MI RE	1	96.6 (78.3)		
		2	122.9 (113.8)	27.15%	
		3	137.6 (116.2)	42.37%	
		4	138.3 (125.5)	43.11%	
		5	146.2 (137.2)	51.30%	

*Note*: Timepoint 1, the start of the first set; timepoint 2, the end of the first set; timepoint 3, the end of the second set; timepoint 4, the end of the third set; timepoint 5, the end of the fourth set. *p < 0.05; **p < 0.01; ***p < 0.001.

Abbreviations: BFR, blood flow restriction; M, mean; MI, moderate intensity; RE, resistance exercise; RF, rectus femoris; SD, standard deviation; VL, vastus lateralis.

### 3.2 Δ MDF

For rectus femoris, the linear mixed model analysis revealed no significant interaction between time and condition (*F*(1, 24) = 1.27, *p* = 0.27), a significant main effect of time (*F*(1, 24) = 4.62, *p* = 0.042) and no significant main effect for condition (*F*(1, 24) = 0.11, *p* = 0.74) (Figure 1C).

For VL, the linear mixed model analysis revealed no significant interaction between time and condition (*F*(1, 26) = 2.52, *p* = 0.12), a significant main effect for time (*F*(1, 26) = 49.90, *p* < 0.001) no significant main effect for condition (*F*(1, 26) = 0.083, *p* = 0.77) (Figure 1D). The means and standard deviation for rectus femoris Δ MDF and vastus lateralis Δ MDF are presented in Table 3 below.

**TABLE 3:** Mean (SD) Δ MDF.

Muscle	Condition	Timepoint	*M* (*SD*) (*Hz*)
RF	BFR RE	1	55.50 (5.93)
		2	54.18 (9.27)
	MI RE	1	56.19 (8.31)
		2	51.46 (5.38)
VL	BFR RE	1	59.99 (14.84)
		2	52.44 (12.57)
	MI RE	1	60.72 (14.06)
		2	48.79 (13.66)

*Note*: Timepoint 1, the start of the RE protocol; Timepoint 2, the end of the RE protocol.

Abbreviations: BFR, blood flow restriction; M, mean; MI, moderate intensity; RE, resistance exercise; RF, rectus femoris; SD, standard deviation; VL, vastus lateralis.

### 3.3 Exploratory analyses

#### 3.3.1 Effect of sex

During BFR RE, females showed lower rectus femoris and vastus lateralis Δ RMS compared to males (rectus femoris Δ RMS 31.98 ± 47.01% vs. 90.46 ± 122.67%, *g* = 0.63; vastus lateralis Δ RMS: 24.54 ± 22.03% vs. 46.91 ± 59.28%, *g* = 0.50, females and males, respectively). Females demonstrated larger Δ MDF changes in rectus femoris and similar Δ MDF changes in vastus lateralis compared to males (rectus femoris Δ MDF: −5.25 ± 7.68% vs. −0.46 ± 9.94%, *g* = 0.54; vastus lateralis Δ MDF: −11.29 ± 10.11 vs. −12.17 ± 10.56, *g* = 0.09, females and males, respectively).

During moderate intensity RE, females showed lower rectus femoris and vastus lateralis Δ RMS compared to males (rectus femoris Δ: RMS 49.93 ± 42.25% vs. 119.81 ± 130.61%, *g* = 0.72; vastus lateralis Δ RMS: 11.82 ± 18.42% vs. 67.84 ± 47.17%, *g* = 1.56, females and males, respectively). Females demonstrated smaller Δ MDF changes in rectus femoris and similar Δ MDF changes in vastus lateralis compared to males (rectus femoris Δ MDF: −0.93 ± 14.16% vs. −11.61 ± 11.32%, *g* = 0.83; vastus lateralis Δ MDF: −16.84 ± 8.62% vs. −22.20 ± 16.25, *g* = 0.41, females and males, respectively).

#### 3.3.2 Effect of EIH

We defined EIH responders based on minimum detectable change cutoff in PPT (4.27 N/cm^2^) obtained from previous studies in a pain‐free population (Alsouhibani et al., 2019; Walton et al., 2011). Participants who demonstrated an increase in PPT greater than 4.27 N/cm^2^ after exercise were recognized as EIH responders.

With BFR, there were 11 EIH responders and three nonresponders. During BFR RE, EIH responders showed greater rectus femoris and vastus lateralis Δ RMS compared to nonresponders (rectus femoris Δ: RMS 81.69 ± 95.80 vs. 2.80 ± 2.72, *g* = 0.90; vastus lateralis Δ RMS: 46.36 ± 43.77 vs. −3.25 ± 19.90, *g* = 1.21, EIH responders and nonresponders, respectively). EIH responders demonstrated similar Δ MDF changes compared to nonresponders (rectus femoris Δ MDF: 1.82 ± 9.29 vs. 5.95 ± 8.04, *g* = 0.45; vastus lateralis Δ MDF: 12.34 ± 10.64 vs. 9.50 ± 8.18, *g* = 0.28, EIH responders and nonresponders, respectively).

With MI, there were six EIH responders and eight nonresponders. During moderate intensity RE, EIH responders demonstrated greater rectus femoris Δ RMS (119.53 ± 139.64 vs. 58.88 ± 53.51, *g* = 0.61) and greater rectus femoris Δ MDF (11.38 ± 12.47 vs. 2.43 ± 13.73, *g* = 0.68) and similar vastus lateralis Δ RMS (50.22 ± 63.27 vs. 32.04 ± 27.32, *g* = 0.40) and vastus lateralis Δ MDF (22.45 ± 7.42 vs. 17.32 ± 15.88, *g* = 0.39) compared to nonresponders.

## 4 DISCUSSION

The overall purposes of this study were to compare the effect of low intensity BFR RE versus conventional moderate intensity RE on muscle activity in a single session effort‐matched RE protocol and explore the interplay of muscle activity, sex, and EIH in this context. We found that both modalities, BFR and conventional moderate intensity RE, were capable of increasing muscle activity and inducing muscle fatigue in response to an effort‐matched protocol. Contrary to our hypothesis, no significant differences in Δ RMS and Δ MDF were found between BFR and moderate intensity RE conditions. Our exploratory analysis found that female consistently showed lower muscle activity increase in both exercise conditions, with more heterogeneous findings in MDF changes. We also found greater Δ RMS in EIH responders compared to nonresponders in BFR RE and moderate intensity RE, which may indicate a critical role of muscle activity in RE‐related pain modulation. Furthermore, while inconsistent Δ MDF were noted in EIH responders versus nonresponders during BFR RE, greater Δ MDF was found in EIH responders compared to nonresponders in moderate intensity RE, which may suggest the involvement of high threshold motor units in mediating EIH in moderate intensity RE.

When compared to studies with similar protocols (did leg press at 30% 1RM with BFR), similar RMS activity was noted in the current study and previous research (Wilson et al., 2013). However, higher RMS at the end of the final set was found in our study possibly due to the use of exercise to volitional fatigue in our study versus the use of a fixed repetition protocol (i.e., 30‐15‐15‐15) applied in theirs.

One unique contributions of the present study is that we compared changes in muscle activity and median frequency between low intensity BFR RE and conventional moderate intensity RE, while the majority of available evidence has either compared low intensity BFR RE (e.g., <30% 1RM) versus conventional high intensity RE (e.g., >70% 1RM) or low intensity BFR RE versus intensity‐matched conventional low intensity RE (e.g., <30% 1RM) (Buckner et al., 2019; Freitas, Galletti, et al., 2020; Freitas, Miller, et al., 2020; Sousa et al., 2017; Yasuda et al., 2014, 2015). The conditions compared in the present study are in line with current practice recommendations and have the most external validity for clinical practice (Jimeno‐Almazán et al., 2022; Kim et al., 2019). Specifically, conventional moderate intensity RE is more efficient to achieve muscle adaptation compared to low intensity RE, and it is safer and more feasible for load‐compromised individuals (e.g., such as those undergoing postoperative rehabilitation) compared to high intensity RE (Melo et al., 2022; Miyachi et al., 2004). Furthermore, emerging evidence shows that low‐to‐moderate intensity RE (30%–50% 1RM) has widespread utility across individuals with fibromyalgia, multiple sclerosis, and knee osteoarthritis, and can elicit positive strength and functional outcomes without pain exacerbation (Gowans & deHueck, 2004; Halabchi et al., 2017; Messier et al., 2021; Nelson, 2015). Thus, the comparisons between low intensity BFR RE and moderate intensity RE are more meaningful from a clinical perspective.

High degree of muscle fiber recruitment, especially type II fibers, has been postulated to be one of the key mechanisms underlying muscle hypertrophy induced by low intensity BFR RE (Abe et al., 2012; Farup et al., 2015; Takarada et al., 1985; Wernbom et al., 2008) and exercising to fatigue can enhance the recruitment of both type I and type II fibers (Suga et al., 2012; Takada et al., 1985; Wernbom et al., 2008). Therefore, the current study used a fatiguing BFR RE protocol instead of the common arbitrary and frequently used four sets 75‐repetition BFR protocol (i.e., 30‐15‐15‐15) (Patterson et al., 2019). In addition, unlike other fatiguing protocols that exercising to task failure at each set, the participants in this study only worked to failure at the last set. From an exercise training perspective, this method could combat the dramatic lower total exercise volume observed in other fatiguing protocols (Song et al., 2022; Wernbom et al., 2009) while ensuring relatively high training volume and exercise‐contraction duration, which is beneficial for muscle adaptation (Narici et al., 1989; Schoenfeld et al., 2019).

Contrary to our hypothesis, both exercise modalities increased muscle activity over time and ended at a similar level. However, only in the BFR condition, RMS activity progressively increased over sets, while the maximum myoelectric activity was reached before the end of the last set in moderate intensity condition. This pattern is in line with previous studies (Fatela et al., 2018; Loenneke et al., 2015) and supports the notion that different muscle fiber recruitment strategies may be involved in the two exercise modalities (Fatela et al., 2018). A potential explanation for different recruitment strategies in BFT versus moderate intensity RE is based on the contention that afferent input may affect alpha motor neuron excitability. Current studies have postulated that when exercising with BFR, the applied circumferential pressure may disturb the balance of excitatory and inhibitory inputs onto motor neurons and result in decreased Hoffman‐reflex (H‐reflex), which primarily recruit smaller motoneurons, and no changes on F‐wave, which preferentially recruit larger motoneurons (Wernbom & Aagaard, 2020). Thus, the high‐threshold units may be recruited earlier than normal to maintain force output and avoid failure during BFR exercise (Loenneke et al., 2011; Yasuda et al., 2010).

By analyzing MDF of the EMG power in both conditions, our results suggest that, when exercising to volitional failure, both RE protocols induce similar levels of neuromuscular fatigue. The changes noted in MDF during BFR RE in the current study are partially in line with previous studies (Fatela et al., 2016, 2018), in which a 4‐set 75‐repetition protocol was used, and the reduction in MDF was limited to rectus femoris but no other quadriceps muscles (i.e., vastus medialis). The disparity could be partially due to the differences in the RE protocol (leg press vs. open chain extension, and exercise to failure vs. fixed volume) and potentially supports the application of current BFR protocols in muscle strength and conditioning.

Our exploratory analyses indicate that the myoelectrical activity might be affected by sex in BFR and moderate intensity RE while exercising to task failure, with females showed less sEMG amplitude increase compared to males. This sex difference is in line with a previous study, which involved similar settings as the present one. In that study, Freitas et al. compared the sEMG amplitude changes in response to low intensity (30% 1RM) leg press exercise with and without BFR (50% LOP) using a 30‐15‐15‐15 protocol (Freitas, Miller, et al., 2020). Similar to the present study, they found consistent greater RMS increase in males compared to females in both conditions (Freitas, Miller, et al., 2020). One possible explanation for this sex difference could be the larger cross‐sectional area of type II muscle fiber in the quadricep muscles in young men than it in women (Staron et al., 2000). Future studies are needed to explore whether differences in muscle activity may mediate sex‐based differences in RE outcomes.

In this study, we found a significantly greater amount of muscle activity in EIH responders compared to nonresponders in BFR RE and moderate intensity RE. These findings may indicate the importance of muscle activity in RE‐mediated pain modulation and may explain why consistent and profound EIH is commonly observed following both, high intensity exercise or prolonged low intensity tasks (Hoeger Bement et al., 2008; Koltyn, 2000; Naugle et al., 2012). When exercising to volitional fatigue, motor unit recruitment strategies are altered, reflected as increased RMS and decreased MDF (Fallentin et al., 1993; Mujumdar et al., 2022). Not surprisingly, EIH responders also showed greater MDF decrease compared to nonresponders in moderate intensity RE. However, inconsistent MDF changes were noted between EIH responders and nonresponders while exercising with BFR. This may suggest different mechanisms are involved in pain modulation between the two modalities.

This study also have several limitations. Relatively small sample sizes were used for the two exploratory aims. Large scale studies are needed to confirm the sex difference on muscle activity and the role of muscle activity on EIH. Furthermore, the leg press exercise also involved hip muscles recruitment, which might interfere the consistency of muscle recruitment patterns, especially while exercising towards the task failure. To minimize the involvement of glutes muscles and maximize the dominance of quadriceps muscles during the exercise, participants were instructed to place their dominant foot at the lower edge of the plate to reduce the hip muscles engagement during the exercise. Lastly, though the PPT testing procedure was conducted strictly followed the suggestions from previous reliability studies, the awareness of the exercise condition might rise the possibility of subjective bias from the tester.

## 5 CONCLUSION

Low intensity BFR RT‐induced comparable muscle activity compared to conventional moderate intensity RT while exercising to task failure. Additionally, there is a sex effect on muscle activation, with female participants showed lower level of muscle activation compared to male subjects in both exercise conditions. Our subgroup analysis on pain modulation responses indicates a close relationship between muscle activity and exercise‐induced hypoalgesia in both conditions.

## FUNDING INFORMATION

This study is partially supported by the NYU Steinhardt Doctoral Research and Travel Grant to Jinghui Yang.

## CONFLICT OF INTEREST STATEMENT

All authors declare that they have no conflicts of interest.

## 6 ETHICS STATEMENT

This study was approved by the New York University Institutional Review Board (IRB‐FY2019‐3039). All experimental precedures were strictly conducted following the approved IRB. All participants received written information on the course and purpose of the research and gave their written consent prior to data collection.

## Data Availability

The data that support the findings of this study are available from the corresponding author upon reasonable request.
